# The impact of endemic and epidemic malaria on the risk of stillbirth in two areas of Tanzania with different malaria transmission patterns

**DOI:** 10.1186/1475-2875-5-89

**Published:** 2006-10-17

**Authors:** Ulrika Uddenfeldt Wort, Ian Hastings, TK Mutabingwa, Bernard J Brabin

**Affiliations:** 1 Division of International Health (IHCAR), Karolinska Institutet, Stockholm, Sweden; 2 Child and Reproductive Health Group, Liverpool School of Tropical Medicine, Pembroke Place, Liverpool L3 5QA, UK; 3 Gates Malaria Partnership, London School of Hygiene and Tropical Medicine, London, UK; 4 National Institute of Medical Research, Dar es Salaam, Tanzania; 5 Emma Kinderziekenhuis, Academic Medical Centre, University of Amsterdam, The Netherlands; 6 Royal Liverpool Children's Hospital NHS Trust, Alder Hey, Liverpool, UK

## Abstract

**Background:**

The impact of malaria on the risk of stillbirth is still under debate. The aim of the present analysis was to determine comparative changes in stillbirth prevalence between two areas of Tanzania with different malaria transmission patterns in order to estimate the malaria attributable component.

**Methods:**

A retrospective analysis was completed of stillbirth differences between primigravidae and multigravidae in relation to malaria cases and transmission patterns for two different areas of Tanzania with a focus on the effects of the El Niño southern climatic oscillation (ENSO). One area, Kagera, experiences outbreaks of malaria, and the other area, Morogoro, is holoendemic. Delivery and malaria data were collected over a six year period from records of the two district hospitals in these locations.

**Results:**

There was a significantly higher prevalence of low birthweight in primigravidae compared to multigravidae for both data sets. Low birthweight and stillbirth prevalence (17.5% and 4.8%) were significantly higher in Kilosa compared to Ndolage (11.9% and 2.4%). There was a significant difference in stillbirth prevalence between Ndolage and Kilosa between malaria seasons (2.4% and 5.6% respectively, p < 0.001) and during malaria seasons (1.9% and 5.9% respectively, p < 0.001). During ENSO there was no difference (4.1% and 4.9%, respectively). There was a significant difference in low birthweight prevalence between Ndolage and Kilosa between malaria seasons (14.4% and 23.0% respectively, p < 0.001) and in relation to malaria seasons (13.9% and 25.2% respectively, p < 0.001). During ENSO there was no difference (22.2% and 19.8%, respectively). Increased low birthweight risk occurred approximately five months following peak malaria prevalence, but stillbirth risk increased at the time of malaria peaks.

**Conclusion:**

Malaria exposure during pregnancy has a delayed effect on birthweight outcomes, but a more acute effect on stillbirth risk.

## Background

Stillbirth is an important indicator of the quality of obstetric care. The highest rates of stillbirth occur in developing countries, especially sub-Saharan Africa, and averages 20-40/1000 births [1]. Malaria can cause serious complications in pregnancy, such as anaemia, low birthweight, pre-term delivery, and maternal mortality. The reductions in birthweight occur particularly in primigravidae in areas with an endemic malaria transmission pattern [2-7].

The impact of malaria on the risk of stillbirth and neonatal mortality is still under debate. Malaria could increase stillbirth risk through low birthweight, foetal anaemia or preterm delivery [8]. Neither McGregor et al., in the Gambia [3], nor Anagnos et al. in Zaire [9], detected any statistical association between stillbirth and placental malaria. McGregor et al. reported some seasonal differences in stillbirth rates, with the lowest rate occurring during the three months of the late dry season when placental malaria prevalence was low. This difference occurred across all parities. Conversely Okoko et al., also in the Gambia, observed a two-fold increased risk of stillbirth among mothers with malaria-infected placenta [10]. Recently, van Geertruyden et al. reached the same conclusion based on a meta-analysis of 17 cross-sectional studies mostly from Africa [1]. They also concluded that the foetal mortality rate was doubled in malaria endemic countries (40.1/1000, 95% CI; 32.1–48.0), compared to non-endemic countries (20.0/1000, 95% CI; 13.2–26.8). These studies were hospital based, parity was not taken into account and most were from highly malarious areas.

Stillbirth is common in areas with unstable malaria [11-13] and amongst refugees [14]. Newman et al. reported a seven-fold increased risk of stillbirth in association with placental parasitaemia in areas with unstable malaria transmission [15]. All of these studies were confounded by non-malaria related causes of stillbirth, which may relate to history of recurrence [16]. Methodologies to estimate the malaria-attributable component are difficult to establish. In view of the association of malaria with low birthweight and the El Niño southern climatic oscillation (ENSO) [57], this natural phenomenon facilitates estimation of malaria attributable stillbirths related to peak periods of malaria exposure, or to epidemic malaria, which can occur at these times.

The aim of the present analysis was to determine comparative changes in stillbirth and low birthweight prevalence between two areas of Tanzania with different malaria transmission patterns in order to estimate seasonal changes and the malaria attributable component.

## Methodology

### Study area Kagera (Ndolage mission hospital)

This region is situated in the north-west corner of Tanzania, and is an area with endemic malaria and strong seasonality. Kagera is one of the poorest regions in Tanzania. Most women of reproductive age are subsistence farmers who cultivate plantains, maize, cassava, rice and millet. Ndolage hospital, in Muleba district (formerly Bukoba rural district) is situated 60 km south of Bukoba town, at an altitude of 1,600 metres above sea level. It is a mission hospital with approximately 1,400 deliveries a year, contributing to approximately 45% of the deliveries in the hospital catchment area. The state-run antenatal clinics do not provide antimalarial prophylaxis, but those run by the churches sometimes do (chloroquine 300 mg base once weekly at the time of the study). The effectiveness of chloroquine prophylaxis was greatly limited by poor compliance and high levels of chloroquine resistance [18].

Retrospective data was collected for deliveries at Ndolage hospital for the years 1994–1999. Information from delivery books was transcribed to data sheets and entered into an SPSS data file. Data on stillbirth, birthweight, parity and twin birth were collected. Records included both macerated and fresh. The data on peripheral blood smear examination for malaria parasites was obtained from laboratory record books for the same period of deliveries. Malaria smears were routinely taken from all in and out-patients with malaria-like symptoms who visited the hospital. Quality control of malaria microscopy was not available, but the hospital technicians were experienced malaria microscopists. Data on all diagnosed malaria cases admitted to the paediatric ward was collected. Data on rainfall was available locally from Bukoba Meteorological Station.

**Figure 1:**
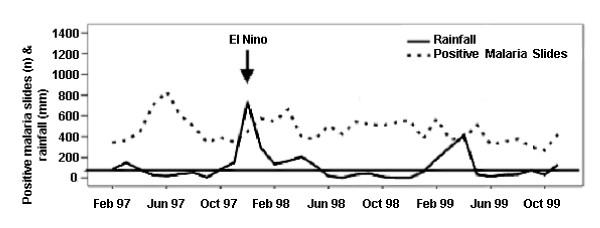
Positive malaria slides at Ndolage hospital and rainfall in Bukoba District (1994–1999).

Malaria-related climatic effect on the risk of low birthweight was considered to be greatest a number of months into a peak period of malaria transmission, as a longer duration of malaria exposure during pregnancy would increase the risk of birthweight reduction. It was predicted this effect would occur three months after the malaria peak and that it would last 5 months. This approximation was made following an analysis of data from an earlier study in Kagera region [7,17]. It was assumed that the increased risk of stillbirth would last as long as the malaria season lasted. The rainy season was defined as rainfall >80 mm/month. In some years a clear seasonal rainfall pattern was observed even if monthly values did not fall below 80 mm. The malaria season was defined as a change in prevalence from baseline value which averaged >150 positive malaria slides per month (all ages) (Figure 1). This represented a weighted average estimate for positive slides. Seasonal trends were assessed. At the time of the study, treatment failures with chloroquine (25 mg/kg) were 50% at day 14 following treatment [18].

To compare the results from the two study areas, the study period was divided into three groups in relation to seasonal intensity of malaria transmission: between malaria seasons, during malaria seasons (stillbirths), in relation to malaria seasons (low birthweight) and El Niño Southern Oscillation (ENSO). This was one of the worst El Niño ever recorded and lasted between November 1997 and August 1998. The El Niño rains in Kagera came with strong winds, which uprooted plantain the staple food leading to increased famine, especially as El Niño was followed by a subsequent drought.

### Study area Morogoro (Kilosa district hospital)

**Figure 2:**
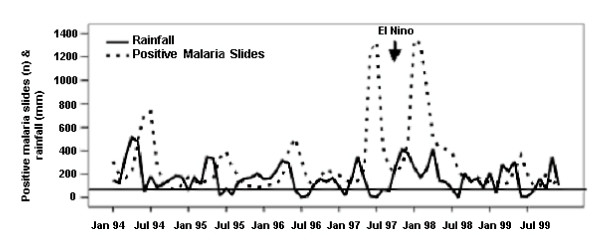
Positive malaria slides at Kilosa hospital and rainfall in Kilosa District (1997–1999).

This region is situated in central Tanzania and is an area with holoendemic malaria transmission with seasonal peaks [19,20]. Apart from the Uluguru mountain ranges, most (80%) of the region is flat, with a risk of standing water following the rainy period. Most women are subsistence farmers who cultivate maize, cassava, rice and shogum. Kilosa hospital has 150 beds and 1200–1400 deliveries a year representing approximately 35% of deliveries in the catchment area. Data were available from Kilosa Hospital for the years 1994–1999 and from the local meteorological station. Data on peripheral blood smear examination for malaria parasites was available from laboratory books from 1997–1999. The staff at the nine antenatal clinics visited, and the doctor in charge of Kilosa hospital stated that malaria prophylaxis, or preventive antimalarial treatment was not given routinely. Data on clinically diagnosed malaria cases from the paediatric ward was also collected to confirm malaria seasons. The rainy season was as defined in Kagera region. The malaria season in Kilosa was defined similar to that in Ndolage hospital, as a change in prevalence from a baseline value which for Kilosa was >450 positive malaria slides per month (Figure 2). This was the weighted average estimate for this area [7]. For Kilosa, the different malaria seasons were defined as in Kagera. ENSO in Kilosa also came with heavy rainfall, but did not cause any malaria epidemic. At the time of the study clinical chloroquine treatment failures (all ages) at day 14 following treatment were 72% [18].

Data was entered into SPSS^®^. A logistic regression model was used, with separate analysis for low birthweight and stillbirth). Only one predictor variable was used, that of risk period (as defined above) as a fixed factor. The reference category was deliveries/stillbirths that occurred outwith the defined risk periods. Categorical data were evaluated using chi-square tests. A two-sided p value of < 0.05 was considered statistically significant. Low birthweight was defined as less than 2,500 grams. Twin births and stillbirths were excluded in the low birthweight calculations as their inclusion gave skewed results. Twin births were excluded in the stillbirth calculations for the same reason.

These studies received ethical approval by the Ethical Committee at the National Institute for Medical Research in Tanzania. Permission to undertake the study was granted by the Evangelical Lutheran Church of Tanzania (Ndolage hospital) and the District Medical Officer in charge of Kilosa hospital.

## Results

Descriptive data on low birthweight and stillbirth for the two study areas are summarised in Table 1. There was a significantly higher prevalence of low birthweight in primigravidae compared to multigravidae in both study areas (Ndolage, odds ratio (OR) = 2.0; 95% CI, 1.7–2.3; Kilosa, 2.7; 2.4–3.1). Primigravidae from Kilosa showed significantly higher low birthweight prevalence compared to Ndolage (p < 0.001). There was also significantly higher stillbirth prevalence in Kilosa, with a trend to higher prevalence with increasing gravida.

**Table 1:** Birth outcomes from Ndolage and Kilosa hospitals, 1994–1999

**Hospital**	**Sample size**	**Proportion deliveries in first pregnancy **(95% CI)	Prevalence low birthweight* (95 % CI)			**Prevalence all stillbirths **(95 % CI)		
			
			All	PG	MG	All	PG	MG
**Ndolage**	7,825	36.8 (35.8–37.9)	11.9 (11.2–12.6)	16.2 (14.9–17.6)	9.4 (8.6–10.2)	2.4 (2.0–2.7)	1.7 (12-2.1)	2.6 (2.1–3.0)
**Kilosa**	7,350	39.7 (38.6–40.8)	17.5 (16.6–18.4)	25.5 (23.9–27.1)	12.2 (11.2–13.2)	4.8 (4.3–5.3)	4.2 (3.4–4.9)	5.2 (4.5–5.8)

* twins and stillbirth excluded, risk period for low birthweight is calculated to start 3 months after the peak malaria period and to last for 5 months.

### Kagera region (Ndolage hospital)

The rains in Kagera were seasonal (Figure 1). All rainy periods were followed by malaria seasons, with one exception (July-December 1994). For two periods the short and the long rains merged into long rainy periods both lasting 9 months. The first was from September 1995-May 1996 and the average rainfall per month was low. The second lasted from October 1997-June 1998 and was the worst ENSO recorded.

Malaria seasons lasted for 2–4 months, with three exceptions. In April-August 1994 the malaria season lasted for 5 months. It was a recognized heavy malaria season with an average of 488 positive malaria slides per month. In April-September 1995 the season lasted for 6 months but was less impressive (250 slides/month). The heaviest malaria season, which was epidemic, was during the ENSO months between November 1997 and August 1998. It lasted 10 months with 631 positive malaria slides per month. June-September 1997 was another exceptional malaria period (820 positive malaria slides/month), although short (4 months) and not preceded by high rainfall. Between these rainy seasons, there was a considerably smaller malaria risk. Diagnosed malaria in children in the paediatric ward and positive malaria slides for the whole population showed good temporal correspondence.

Between 1994 and 1999 there were 7,825 singleton live and stillbirths at Ndolage hospital, of which 0.2% had missing data. The proportion of primigravidae births (36.8%) was above that expected for a rural hospital in a developing country [21] (Table 1). The period for highest risk of low birthweight in primigravidae compared to multigravidae was calculated as commencing three months after the major malaria peak and lasting for five months [7]. Two major risk periods for increased prevalence of low birthweight in primigravidae were recognized, one was in relation to the heavy malaria season April-August 1994 (OR = 1.7; 95% CI, 1.2–2.5, p = 0.007) and the other one was in relation to ENSO (OR = 1.7; 95% CI, 1.1–2.6, p = 0.013). ENSO was the only identified risk period for increased prevalence of stillbirth (OR = 1.9; 95% CI, 1.3–2.9, p = 0.001), (Table 2).

**Table 2:** Logisitic regression of risk of stillbirth in all deliveries and of low birthweight in primigravidae, 1994–1999; one predictor was fitted in each analysis i.e. risk period as defined in the main text. Overall significance of risk period in Ndolage for stillbirth and LBW was p = 0.002 and p = 004 respectively, and for Kilosa was p = 0.55 and p = 0.25 respectively

**Malaria season**	**OR for stillbirth**		Risk period*	OR for lbw in PG*	
			
	**Ndolage**	**Kilosa**		**Ndolage**	**Kilosa**
**Between malaria seasons**	Reference	Reference	**Between risk periods**	Reference	Reference
**Dec 93-Feb 94**	0.7 (0.3–1.6)	N/A	**April 94-Aug 94**	1.1 (0.8–1.7)	N/A
**April 94-Aug 94**	1.3 (0.8–2.2)	N/A	**Sep 94-Jan 95**	1.7 (1.2–2.5)^§^	N/A
**April 95-Sep 95**	1.3 (0.8–2.1)	N/A	**Sep 95-Jan 96**	1.4 (0.9–2.0)	N/A
**April 96-July 96**	0.6 (0.3–1.4)	N/A	**Aug 96-Dec 96**	0.7 (0.4–1.2)	N/A
**Oct 96-Jan 97**	0.4 (0.1–1.1)	N/A	**Feb 97-June 97**	1.0 (0.6–1.5)	N/A
**Jun 97-Aug 97**	0.4 (0.1–1.2)	1.2 (0.7–2.1)	**Sep 97-Jan 98**	0.6 (0.3–1.0)	1.0 (0.7–1.4)
Nov 97-Aug 98**	1.9 (1.3–2.9)^†^	0.8 (0.6–1.2)	**April 98-Aug 98**	1.7 (1.1–2.6)^§^	0.8 (0.6–1.2)
**May 99-July 99**	1.6 (0.9–2.9)	0.9 (0.5–1.6)	**Aug 99-Dec 99**	1.2 (0.8–1.7)	1.3 (0.9–2.0)

* twins and stillbirth excluded, risk period for low birthweight is calculated to start 3 months after the peak malaria period and to last for 5 months.

**ENSO, ^†^p = 0.001, ^§^p = 0.01

For the years 1994–1999, the prevalence risk of stillbirth was compared between and during normal malaria seasons and during ENSO. The prevalence of low birthweight between and in connection with malaria seasons and in connection with the two heavy malaria seasons 1994 and 1997–1998 (ENSO) was also compared. Low birthweight prevalence in primigravidae did not differ greatly between periods between or in relation to normal malaria transmission (14.3% and 14.7% respectively), but significantly increased in primigravidae during 1994 and ENSO to 22.0% (p = 0.002). Stillbirth prevalence increased during ENSO for both primigravidae and multigravidae, although this was significant only for multigravidae (p = 0.018) and only for those with birthweight 2.5 kg and above (p = 0.02). Macerated stillbirth prevalence was 50%, fresh stillbirth 21% and undefined stillbirths 29%. Regression analysis for stillbirth risk for the individual months for combined years was significant for October (2.6; 95% CI, 1.1–6.1).

### Morogoro region (Kilosa hospital)

Rainfall showed a seasonal pattern (Figure 2) which was not as marked as in Kagera. The short and long rains often merged into a period lasting approximately 4 months, with 400–600 mm rainfall. As in the Kagera, October 1997-May 1998 was an exceptionally heavy rainy season (1884 mm).

Malaria data was available only from April 1995 (diagnosed malaria cases in the paediatric ward) and from January 1997 (positive malaria slides). All rainy periods from April 1995 were followed by malaria seasons, although they were more difficult to define compared to those in Kagera. Malaria increased between May-August 1997 despite little preceding rain. ENSO (October 1997-May 1998) was followed by a malaria season of the same magnitude as in Kagera(Figure 1). During all these periods, including ENSO, there was a high incidence of malaria with 600–900 positive malaria slides per month. Diagnosed malaria cases in the paediatric ward and positive malaria slides for the whole population showed good temporal correlation.

There were 7,350 singleton live and stillbirths at Kilosa hospital during 1994–1999, of which 1.4% had missing data (Table 1). The prevalence of low birthweight among primigravidae was 25.5% (95% CI, 23.9–27.1), and among multigravidae 12.2% (95% CI, 14.9–17.6). The proportion of primigravidae births was 39.7%. As for Kagera, the peak risk period for increased low birthweight in primigravidae and multigravidae was estimated as commencing three months after the major malaria peak and lasting for five months [17]. The stillbirth rate was significantly lower in primigravidae (4.2%; 95% CI, 3.4–4.9) than in multigravidae (5.2%; 95% CI, 4.5–5.8, p = 0.049). Forty-four percent of stillbirths were low birthweight. Macerated stillbirth prevalence was 49%, fresh stillbirth 32% and undefined stillbirths 19%.

For the years 1997–1999, the prevalence of stillbirth and low birthweight was compared between malaria normal seasons, in connection with malaria seasons, and during and after ENSO. For Ndolage stillbirth and low birthweight prevalence were both significantly increased for all malaria seasons combined when compared to periods between malaria seasons (p = 0.002 and p = 0.004 respectively). For Kilosa there was no significant difference in prevalence of these outcomes for the same comparison. Comparisons of outcomes for specific malaria seasons are listed in Tables 2 and 3.

**Table 3:** Comparison of Ndolage and Kilosa delivery data between, 1997–1999

Malaria transmission level	Location	Stillbirth*			Low birthweight**		
		
		**All**	**PG**	**MG**	**All**	**PG**	**MG**
Between malaria seasons	Ndolage	2.4	1.2	3.2	9.8	14.4	7.1
	Kilosa	5.6	4.6	6.3	15.4	23.0	10.0
	Odds ratio	2.4 (1.7–3.5)	2.9 (1.8–8.5)	2.1 (1.3–3.1)	1.68 (1.36–2.07)	1.8 (1.3–2.4)	1.5 (1.1–2.0)
	p-value	<0.001	<0.001	<0.001	<0.001	<0.001	0.021
Malaria seasons	Ndolage	1.9	2.5	1.4	11.7	13.9	9.8
	Kilosa	5.9	5.1	6.5	16.5	25.2	10.2
	Odds ratio	3.2 (1.7–5.9)	2.1 (0.9–5.3)	4.98 (2.08–11.92)	1.5 (1.2–1.9)	2.1 (1.4–3.0)	1.1 (0.8–1.6)
	p-value	<0.001	>0.05	<0.001	0.001	<0.001	>0.05
ENSO	Ndolage	4.1	3.5	4.5	12.4	22.2	7.4
	Kilosa	4.9	3.3	5.9	14.6	19.8	11.1
	Odds ratio	1.2 (0.8–1.8)	0.9 (0.4–2.1)	1.3 (0.8–2.2)	1.2 (0.8–1.8)	0.9 (0.5–1.5)	1.6 (0.9–2.8)
	p-value	>0.05	>0.05	>0.05	>0.05	>0.05	>0.05

*twins excluded** twins and stillbirth excluded, risk period for low birthweight is calculated to start 3 months after the peak malaria period and to last for 5 months.

Comparing Ndolage and Kilosa for the years 1997–1999, there was a significant difference in stillbirth prevalence between Ndolage and Kilosa between malaria seasons (2.4% and 5.6% respectively, p < 0.001) and during malaria seasons (1.9% and 5.9% respectively, p < 0.001). During ENSO there was no difference, as prevalence increased in Ndolage, (4.1% and 4.9%, respectively) (Table 3). There was a significant difference in low birthweight prevalence between Ndolage and Kilosa between malaria seasons (14.4% and 23.0% respectively, p < 0.001) and in relation to more normal malaria seasons (13.9% and 25.2% respectively, p < 0.001). During ENSO there was no difference as prevalence had increased in Ndolage (22.2% and 19.8%, respectively).

## Discussion

This study reports for the first time a retrospective analysis of seasonal patterns of both low birthweight and stillbirth prevalence in relation to malaria endemicity during high malaria transmission periods and parity. It also reports on the varying risk of low birthweight or stillbirth in different parts of one country with differing malaria transmission patterns and the data coincides for the two areas for the period 1994–1999 (6 years). The analysis is limited as it was retrospective and based on hospital data. Community data on stillbirths would be difficult to obtain without a population-based field study.

The Kagera region, where Ndolage hospital is situated, was hit by an unusual seasonal malaria transmission that led to a severe epidemic during the study period 1994–1999. Standing water after the rainy season became a problem during ENSO 1997–1998 with the consequence of an unusual long and intense malaria transmission season (10 months). During 1994 there was unusually heavy rain and increased *Plasmodium falciparum *positivity with, as a consequence, a prolonged malaria season, but this was not as severe as 1997–1998. Other periods showed seasonal rainfall patterns with malaria seasons mostly lasting 2–4 months.

During the heavy malaria season 1994 and the ENSO low birthweight prevalence in primigravidae was 22.0%, compared to between 14–15 % during the periods between and in relation to malaria seasons. The selective effect of increased low birthweight prevalence on primigravidae suggested that malaria was the primary cause of this differential effect between primigravidae and multigravidae [8]. For the years 1997–1999 and comparing Ndolage and Kilosa there was a significant difference for the risk of low birthweight in primigravidae between and during normal malaria seasons, but a similar prevalence during ENSO. In Morogoro region, where Kilosa hospital is situated, malaria was holoendemic with perennial malaria. As the area is very flat, with a high risk of standing water even after moderate rains, this provided sufficient breeding sites for mosquitoes. Periods were identified when malaria diagnoses were increased and classified these as malaria seasons and considered there was persistent transmission between rainy seasons, due probably to the effects of standing water. Smith et al. carried out a malariometric survey in two villages, situated in the Morogoro region and showed a high prevalence of *P. falciparum *parasitaemia [22]. The estimated mean annual inoculation rate was over 300 infectious bites per person per year with no seasonal fever pattern among children and adults. This constant malaria pressure must have contributed to increasing the risk of low birthweight among primigavidae (25.5%) as first pregnancies are at greatly increased risk of falciparum malaria [2,3,6]. This could relate to the higher risk of stillbirth (4.8%) at Kilosa hospital, which was double that for Ndolage.

Potentially confounding factors which could influence prevalence of stillbirth and low birthweight should be considered [23]. HIV infection has a low prevalence in Kagera region (4.7%), but higher in Morogoro region (9.0%) [24]. HIV incidence is unlikely to alter seasonally, although it is possible that HIV viral load could vary seasonally with associated malaria parasitaemia [25]. The increased incidence of malaria with HIV infection as a combined infection could increase perinatal mortality and stillbirth risk. Syphilis which is an important cause of stillbirth is unlikely to be seasonal although the prevalence in either population is not low (14.9% in Kagera and 12.0% in Morogoro [24]. Nutritional factors could be important and in Kagera crop production during 1997/1998 was good, especially for bananas and sweep potatoes. October was the only month with significantly higher risk for stillbirth, but there is little risk of food shortage then because of harvesting during June-October. In Morogoro the food production dropped by 31% during the ENSO period due to standing water, but the population had good access to staple food [26]. Eclampsia, which is more common in first pregnancies and pre-eclampsia have been associated with malaria exposure in some studies [2,27,28]. Nevertheless the incidence of these conditions is too low to account for the large variation in low birthweight and stillbirth prevalence observed between locations in this analysis.

This data indicates that pregnant women in Kilosa live under a constant high pressure of malaria. During ENSO 1997–98, there was a prolonged malaria season. Despite the immense impact of malaria, there was no significant increased risk of low birthweight or stillbirth during this period. Malaria pressure may have reached a limit in terms of its effect on excess risk of low birthweight in primigravidae, or of stillbirth risk across all parities.

In areas with holoendemic malaria, such as Morogoro, semi-immune women often experience sub-clinical parasitaemias and risk of stillbirth in asymptomatic women may be smaller [8]. In areas with lower endemicity, such as in Kagera, and where host immunity is less developed, the risk of stillbirth would be increased, which was observed at Ndolage hospital. Under these conditions greater fluctuations in stillbirth prevalence would result with seasonal or epidemic malaria, as occurred in Kagera. Women of all parities were at increased risk, although this was highest for multigravidae. Several studies have reported a greater effect of malaria on perinatal mortality in primigravidae than multigravidae [3,5,29,30]. As low birthweight is the single most important risk factor for neonatal mortality [31-34] and the risk of low birthweight is considerably higher in primigravidae, then it might be expected they would experience higher perinatal mortality. This was not observed in the present study in terms of stillbirth risk. The high prevalence is likely to have other causes in addition to malaria. Stillbirths whose birthweight was below 2.5 kg would comprise a group with possibly higher risk of congenital infections such as syphilis, and it was possible many babies died secondary to poor obstetric care.

The high prevalence of anaemia among pregnant women living in malarious areas indicates that foetal anaemia is common, especially in babies whose mothers have concurrent iron deficiency anaemia and high density placental malaria [11]. In these areas average cord haemoglobin may be as low as 13.5 g/dl. Birth in the rainy season has been associated with a 54% incidence of foetal anaemia compared with 34.2% during the dry season [11] and foetal anaemia was associated with increased post-neonatal mortality in low birthweight babies [35]. Placental malaria could increase the risk for stillbirth through maternal and foetal anaemia, and these babies must be more vulnerable to any kind of birth complication (e.g. cord around the neck, or prolonged labour). This could explain the high prevalence stillbirth in Kilosa and an increased malaria related risk of stillbirth during ENSO in Ndolage.

This analysis, shows that malaria exposure during pregnancy had a delayed effect on birthweight outcomes, but a more acute effect on stillbirth risk. The birthweight effect occurred 3–8 months after the peak time of malaria exposure. This is consistent with chronic pregnancy parasitaemias in mid-pregnancy leading to foetal growth restriction, which would be detectable at delivery, a number of months after the peak period of malaria exposure. A recent review of placental malaria has shown that foetal growth restriction is commoner than pre-term delivery with chronic placental infection [36]. This would explain why birthweight was not reduced in primigravidae in relation to the short but intensive malaria period July-August 1997. The effect on stillbirth was greatest during the established malaria epidemic in Ndolage in 1997–1998, suggesting a more acute pathway was involved for stillbirth risk at the time of delivery. This is consistent with the detailed observations of McGregor in the Gambia [3], who reported lower stillbirth rates (all parities) during the three months of the late dry season. In the present study the risk was also lowest during the period between or in connection with ordinary malaria seasons.

This study has also shown that the risk of delivering a low birthweight baby in the first pregnancy increases approximately five months following a malaria epidemic and that the risk of stillbirth almost doubles. The analysis shows a greatly increased low birthweight risk in primigravidae in relation to the heavy malaria season 1994 and ENSO, and also a greatly increased risk of stillbirth in all parities, related to the ENSO of 1997–1998. The areas from which data was collected are considered to experience endemic malaria transmission, but the patterns of exposure during pregnancy and its effects on pregnancy outcome vary to a much greater extent than anticipated across different years.

The use of rainfall for monitoring for malaria early warning has been proposed as a methodology for prediction, prevention and control of malaria epidemics [37,38]. This analysis provides evidence that poor pregnancy outcomes are linked to ENSO years. As a consequence, improved predictions related to climatic factors offers the opportunity for intensifying malaria control measures in order to improve malaria control in pregnancy. This is of particular relevance for communities whose use of malaria control measures in pregnancy is poor. It is essential to provide adequate antimalarial protection to pregnant women, using intermittent preventive treatment and impregnated bed nets, in order to improve pregnancy outcomes. This approach requires adequate monitoring of birthweight indices and stillbirth prevalence [31,40]. Much of this data is routinely collected in hospital facilities but not utilised for surveillance purposes. Improved delivery data acquisition and analysis will be required to successfully harness this approach to strategies to improve malaria control in pregnancy, especially in sub-Saharan Africa.

## Authors' contributions

UW completed the field study and preliminary analysis. IH advised on the study design and statistical analysis. TM assisted with interpretation of data and with writing the paper. BB proposed the study and assisted in its design, analysis, interpretation and in writing the paper.

**Figure 3:**
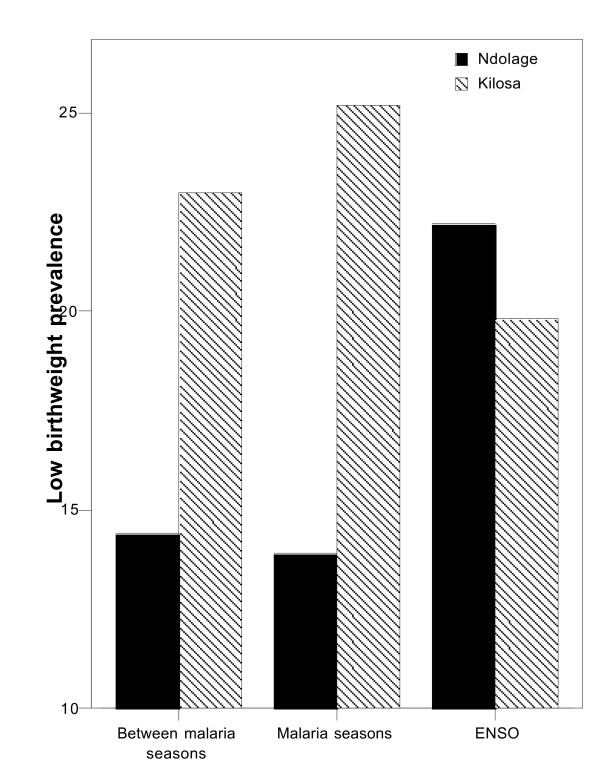
Low birthweight risk during periods with different malaria transmission intensity (1997–1999).

**Figure 4:**
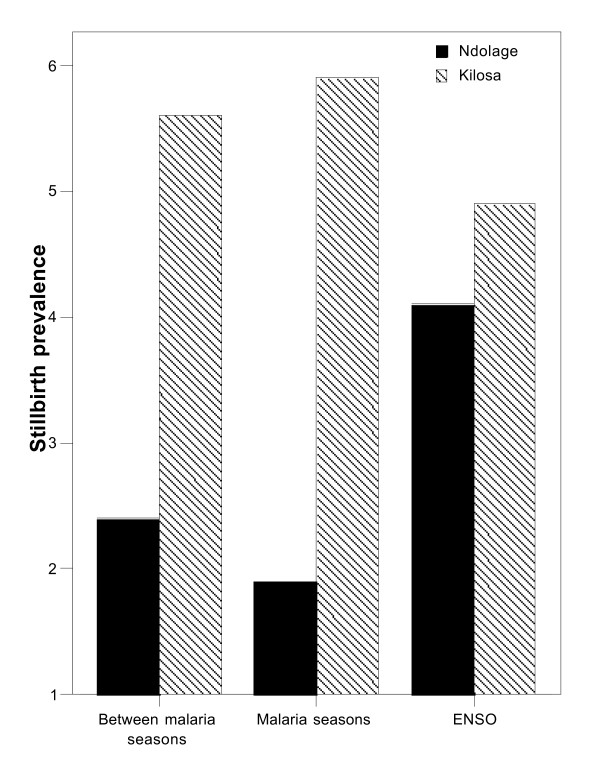
Stillbirth risk during periods with different malaria transmission intensity (1997–1999).
